# Harnessing community conversations for gender-responsive engagement in livestock management in Ethiopia: a methodological reflection

**DOI:** 10.3389/fpubh.2025.1612520

**Published:** 2025-06-25

**Authors:** Mamusha Lemma, Biruk Alemu Gemeda, Theodore Knight-Jones

**Affiliations:** International Livestock Research Institute, Addis Ababa, Ethiopia

**Keywords:** livestock management, gender-responsive approaches, community engagement, community conversations, Ethiopia

## Abstract

**Background:**

Participatory approaches are increasingly employed to design context-specific interventions that are more inclusive, responsive, and effective. The Community Conversation (CC) approach has been tailored to Ethiopia’s livestock management context. As part of the Consultative Group on International Agricultural Research Program on Livestock (CRP Livestock), gender-responsive CC materials on livestock management have been developed and implemented across various rural communities to raise awareness and derive community-led actions. This paper explores how CCs were harnessed through improvisation and provides insights for practitioners to strategically adapt the approach in diverse contexts to foster gender-responsive community engagement.

**Methods:**

Between 2018 and 2019, CCs were conducted at five communities to address different livestock management issues. We selected and trained local facilitators in the CC methodology and documentation process. They used structured facilitation guides and documentation tools to lead conversations. After each session, we held reflection meetings with facilitators to review the process, interpret the discussions, and gain contextual insights. The results were captured in field reports and later analyzed thematically to provide evidence for the approach’s community engagement value.

**Results:**

Findings suggested that CCs hold potential for facilitating collaborative analysis and dialog among rural communities and local service providers regarding gender norms and different livestock management aspects. Gender-inclusive discussions allowed women and men to participate in livestock management decisions. The approach demonstrated adaptability across various contexts and thematic areas. A key strength was its emphasis on collaborative learning and community-driven actions, which helped promote sustained engagement and strengthened partnerships.

**Conclusion:**

The CC approach has shown potential to foster collaboration among rural communities and service providers, enabling them to jointly analyze livestock management challenges and implement locally tailored solutions. Its application in participatory research, training, intervention planning, and partnership building demonstrates its potential to foster collective dialog and action across diverse contexts. Integrating gender perspectives into this approach enhances inclusivity, ensuring that both women and men contribute to decision-making.

## Background

In Ethiopia, livestock is the main livelihood source for smallholder households. However, the livestock sector faces several problems. Among these constraints, herd health management is a major concern for smallholder livestock keepers (1). In pursuit of tailored interventions, as part of the CRP Livestock, the International Livestock Research Institute (ILRI), alongside its local research and development partners, conducted participatory epidemiology and gender analysis to deepen understanding and insights into prioritizing livestock disease constraints, their differential impacts across households, and community awareness and knowledge regarding livestock disease management (2). The study showed significant knowledge and practice gaps among community members about gender and livestock disease management issues. Other studies also showed that animal health service providers had limited gender and community engagement capacity (3). Animal health professionals rarely engage with rural communities to understand their perspectives (4). They often provide community members with prescriptive advice and rarely ask for their views (5). However, influencing community members to change their behavior is a complex and challenging process influenced by many factors (6). Conventional health messaging has a limited impact on shifting community perspectives and changing practices (7).

To address this gap, ILRI and its partners developed community-based herd health management interventions (8). These interventions incorporated gender-responsive and participatory community engagement approaches to facilitate collaborative analysis, learning, and action among rural communities and local service providers (9). Participatory methods are increasingly recommended for raising awareness, promoting knowledge, and driving behavioral change within rural communities through community-driven actions (10). One key method is the CC approach, which provides a platform for rural communities and local service providers to collaboratively analyze challenges, share knowledge, and co-develop solutions (9). This facilitated dialog encourages collaboration, empowerment, and transformation, fostering new ways of thinking and action. In livestock management, the CRP Livestock in Ethiopia adopted the CC approach as a participatory community engagement tool. Gender-responsive CC materials on different aspects of livestock management had been developed and implemented at various intervention sites (11–13).

The approach has yielded promising outcomes in other sectors, including education (14), youth employment and disability (15), rural healthcare (16), harmful traditional practices and health issues (17, 18), infectious diseases (19), and women’s empowerment and gender equality (20). Furthermore, it has been employed as a qualitative research method (21). The CC approach provides a useful way to engage communities and support social and behavioral change, particularly in contexts where awareness, social norms, and community dynamics play an important role.

This paper explores the role of CCs in fostering gender-responsive community engagement within livestock management. It aims to provide methods and insights for practitioners to effectively collaborate with rural communities and adapt the approach effectively in other contexts.

## Methods

### Theoretical foundation and framework

The CC approach of bringing rural communities and local service providers together to leverage local knowledge for context-specific problem-solving resonates with participatory learning and action research approaches. The approach has its roots in participatory methods and learning theories such as appreciative inquiry (22), social learning theory (23), participatory learning and action (24, 25), participatory action research (26), and behavior change model (27).

**Figure 1:**
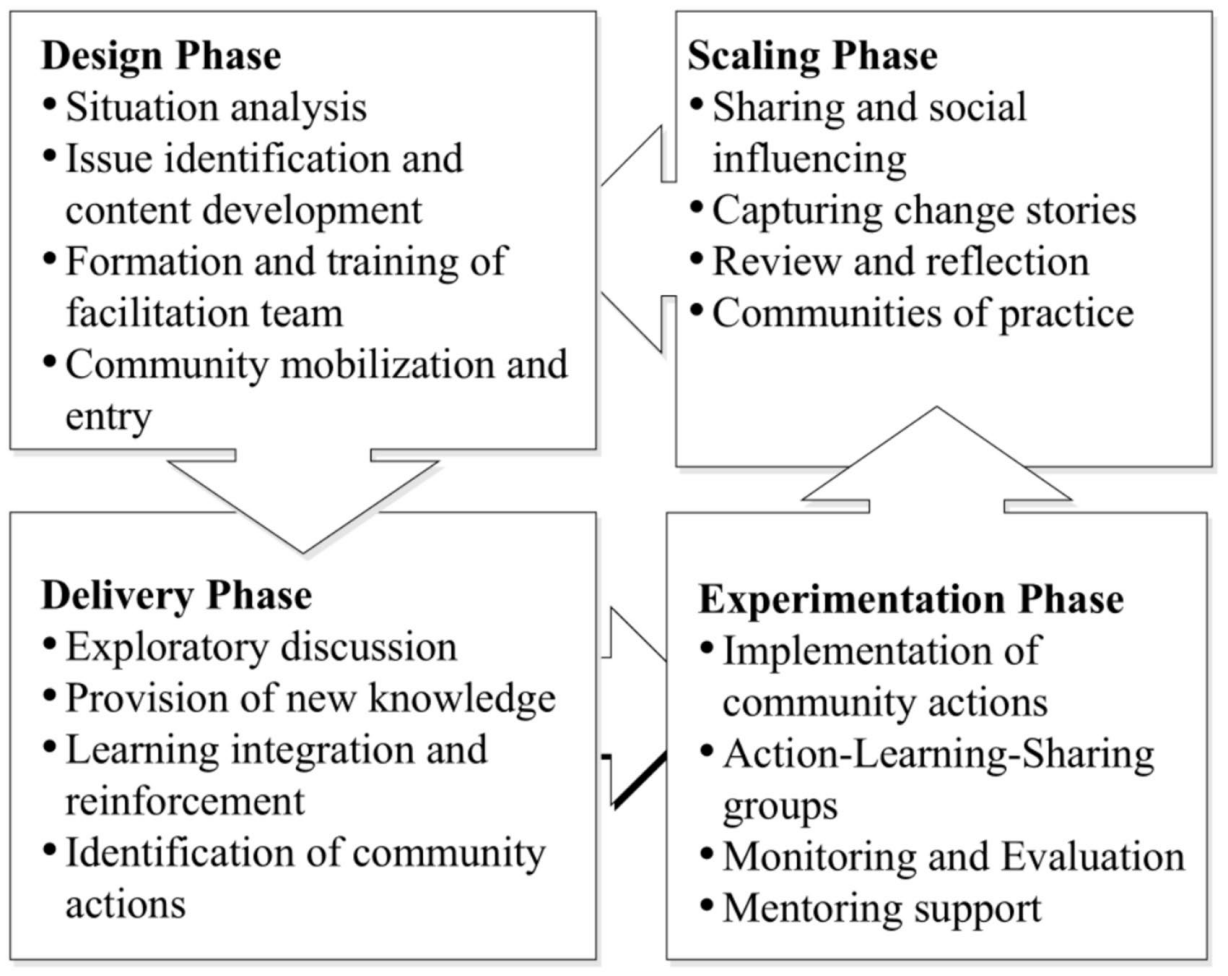
Community conversation (CC) framework implemented in CRP Livestock in Ethiopia.

Leveraging these approaches, we developed a CC framework that describes the iterative phases and activities involved in the process (Figure 1).

The design of the CC process actively involved local research and development partners, who collaborated in developing community engagement materials tailored to local contexts. The delivery of the CC sessions followed an iterative process that encouraged participants to explore diverse perspectives, engage in experiential learning, share knowledge, and collaboratively develop solutions (Table 1). Central to these sessions were community-led actions that drove ongoing learning and sharing. Through experimentation, behavior demonstrations, and technical support from local service providers, communities were able to test and reinforce new practices. According to previous studies (28), the conversations were expected to catalyze broader sharing and social influence within communities through informal networks.

**Table 1:** Community conversation (CC) learning processes as implemented in five CRP Livestock sites in Ethiopia.

Learning steps	Objectives	Methods
Exploration of knowledge, perspectives, and practices	To explore current knowledge, beliefs, and practices of community groups as a foundation for learning	Narrative storytelling, problem scenarios, provocative questioning, picture analysis, role-playing, gender-segregated small group discussions
Introduction of new knowledge	To address knowledge gaps by providing relevant information and skills to improve practices	Interactive communication using illustrations, demonstration of correct behavior
Integration and consolidation of learning	To reinforce new knowledge through communication of key messages for action	Recapping, checking for understanding, sense-making, reinforcing key messages
Identification of community actions	To apply acquired knowledge through community-led initiatives and behavior changes	Action learning and sharing groups, peer action pledge, elder and leader endorsement
Review, reflection, and feedback	To share learning experiences and provide feedback for continuous improvement	Story circles, peer-to-peer interviews

Process monitoring and documentation were central to the successful implementation of the CC approach. Throughout the sessions, participants were actively engaged in reflective discussions. This reflective process allowed participants to discuss the challenges they faced, while also highlighting successful stories that inspired others. By continuously capturing these reflections, the CC approach fostered a dynamic learning environment. This collaborative monitoring process not only strengthened community involvement but also facilitated the sharing of knowledge and solutions tailored to local needs. Local service providers played a key role by offering mentoring and problem-solving support, addressing community-specific challenges, and ensuring that the actions taken were sustainable.

### Description of intervention sites

CCs were conducted in five purposively selected communities in central and southern Ethiopia. These communities were intervention sites under the CRP Livestock. The selection was guided by criteria relevant to the program’s objectives, including high livestock density, representative agroecological zones, and dominant agricultural production systems (29). These characteristics made the sites suitable for piloting and learning from livestock-related interventions. Table 2 provides detailed descriptions of each intervention site.

**Table 2:** Descriptions of intervention sites (45).

Region	District	Population	Communities	Agroecology	Production system
SNNPR	Doyogena	78,600	Hawora Awara Ancha Sadicho	Highland, altitude of 1900–2,300 masl, bimodal rainfall between1200-1600 mm/year, annual temperature between 10–16°C	Mixed crop-livestock dominated by crops, sheep, and cattle
Amhara	Menz Mama	85,130	Key Afer	Moist highland, altitude of 2,500–3,500 masl, bimodal rainfall between1000-1500 mm/year, temperature range of 15–20°C	Mixed crop-livestock system dominated by crops, sheep, and livestock; major crops grown are barley, wheat, and pulses
	Menz Gera	120,470	Sine Amba	Moist highland, altitude of 2,800–3,100 masl, bimodal rainfall between 900–1,000 mm/year, mean annual temperature of 12°C	
Oromia	Yabello	86,400	Dharito	Dry lowland, with an altitude of 350–1800 masl, bimodal rainfall between 300–900 mm, and an annual temperature of 24°C	Pastoralism and agro-pastoralism, dominated by goats, cattle, sheep, and camels

The intervention communities demonstrated socio-cultural, religious, and linguistic diversity, making each context distinct. In Menz, Orthodox Christianity is the dominant religion, while in Doyogena, Protestantism is most prevalent. In contrast, Yabello’s population primarily practices traditional beliefs. The CC approach was customized to fit each community’s unique cultural and social dynamics.

### Participant selection and facilitation process

We formed and trained a team of facilitators drawn from local service providers who were familiar with the communities. The training covered the CC methodology and adult learning principles, blending theoretical sessions with field practice and reflective discussions. To enhance their facilitation skills, the team participated in a mock session before the fieldwork, allowing them to refine their ability to organize, lead, and document community dialogs. We also reviewed the discussion topics with the facilitators to ensure they fully understood the content and could effectively communicate messages to communities. Local service providers played a crucial role in contextualizing discussions, facilitating sessions, documenting field notes, and following up on outcomes. According to previous studies (30), after-event reflections were conducted at the end of each session to capture lessons, insights, and experiences. This has been instrumental in the analysis, validation, and interpretation of conversation results and experiences (31).

The conversations took place in circular seating arrangements, fostering a level playing field and a safe discussion environment. Held in the morning and typically lasting two and a half hours, the sessions took place in open spaces, particularly under a tree or other convenient community locations. They followed a structured format that included an opening session, guided discussions using participatory tools, introduction of new knowledge, and community-led action planning. Each session was formally opened and closed by elders or religious leaders in a culturally appropriate manner, reinforcing community trust, respect for local traditions, and the legitimacy of the process. We aimed for diverse opinions and perspectives in selecting participants to foster a richer dialog, collaborative learning, and community actions. We invited a cross-section of community members, including married women, women-headed households, community leaders, religious leaders, youth, and local service providers, to the CCs.

The CC sessions were conducted as part of a pilot initiative between 2018 and 2019. In the highland sites of Menz and Doyogena, four rounds of sessions were held at monthly intervals in 2018, engaging participants from community-based breeding cooperatives. The sessions were more frequent at the beginning of the intervention and spaced out toward the end to allow participants time to reflect on and practice the actions they had discussed and agreed upon. This period allowed for follow-up on previous sessions and progressive learning through experimentation and sharing. From the outset, we encouraged participants to attend all rounds of the conversations. While the core group of participants remained consistent, a few new individuals replaced some original attendees. This continuity in participation allowed for monitoring and capturing changes and experiences of participants throughout the process.

In 2019, CC sessions were piloted in Yabello to expand the geographic reach of the intervention. Local translators were engaged to facilitate communication across diverse language groups. Due to logistical constraints and the exploratory nature of the engagement in this site, only two rounds of conversations were held on animal welfare and antimicrobial use and resistance. In a world café setup (32), we used separate and mixed-gender groups to explore community perceptions and practices about gender and livestock management issues.

### Data collection and analysis

Although we did not systematically conduct pre-and post-evaluations across all sites to assess impact over time, the CC sessions were supported by a robust and systematic qualitative data collection and analysis process. Note-taking templates were used during sessions to ensure consistent documentation of discussions. This was complemented by direct observation, participant storytelling, and structured reflection exercises. After each round, we engaged facilitators in structured reflection and validation exercises, which formed the primary method of data analysis. This collaborative and iterative process enabled the identification and verification of recurring themes across sessions. Although no data analysis software was used, the iterative and participatory approach contributed to the credibility and contextual relevance of the findings.

Guided by earlier studies (33, 34), we applied thematic analysis to the qualitative data captured in the conversation reports. The analysis was structured around two core questions: (1) how does the CC approach promote gender-responsive engagement and collaboration between rural communities and local service providers? and (2) how do CCs foster social and behavioral change across contexts, and what insights emerge from their processes, methods, and adaptability? The aim was to generate evidence demonstrating the CC approach as an inclusive and participatory method for supporting community-led programs. We reviewed the conversation reports to identify common patterns of experience, which were then organized into key themes and illustrated with direct participant quotes. These themes were further contextualized and validated through relevant literature, strengthening the analytical rigor and practical relevance of the findings.

## Results

### Participant demographics

About 50 participants (40% women) participated in the CC sessions in each community. The average age of participants was approximately 46 years, with ages ranging from 28 to 65. Participation rate was high, with over 80% of invited individuals attending at least three of the four sessions. Women and youth, representing a range of ages and experience levels, were well represented. Most participants had no formal education, while a few had completed primary school. In Yabello, participants were mainly engaged in pastoralism and agro-pastoralism, whereas those in Menz and Doyogena were primarily crop-livestock farmers.

### CC discussion topics and key questions

Between 2018 and 2019, 18 CC sessions were conducted in five communities. These sessions covered diverse topics in livestock management, focusing on gendered perceptions and practices related to livestock ownership and responsibilities, zoonotic risks, animal welfare, and antimicrobial use and resistance (Table 3).

**Table 3:** Main topics discussed during CCs in CRP Livestock sites between 2018 and 2019 in Ethiopia.

Discussion topics	Key questions	Districts	Communities
Gender and livestock management	Gender division of labor and workloads Access to livestock information Access to and control over livestock resources and services	Doyogena, Menz Gera, and Menz Mama	Hawora Awara, Ancha Sadicho, Key Afer, Sine Amba
Gender and zoonotic diseases	Gendered awareness and perceptions of zoonotic diseases Gendered exposure risks of zoonotic diseases Measures to reduce exposure risks	Doyogena, Menz Gera, and Menz Mama	Hawora Awara Ancha Sadicho Key Afer Sine Amba
Animal welfare and livelihoods	Gendered community understanding and perceptions about animal welfare Benefits of animal welfare for animals and people Community needs, constraints, and opportunities for ensuring animal welfare	Menz Gera and Yabello	Sine Amba Dharito
Antimicrobial use and resistance	Gendered community understanding of antimicrobials Information and knowledge sources on antimicrobials Sources and quality of veterinary drugs Gendered use of antimicrobials Community understanding of antimicrobial resistance, its causes, and effects Preventive measures to reduce antimicrobial resistance	Menz Gera and Yabello	Sine Amba Dharito

### Gender dialogs in CC sessions

Gender-related themes were central to all CC sessions, which were intentionally designed to integrate gender perspectives throughout. Approximately 80% of the discussion topics addressed gender aspects, including perceptions, roles, division of labor, participation, decision-making, mobility, workload, and access to services, including information and training. These issues were systematically embedded into session guides and facilitation methods, ensuring that gender was not treated as a standalone issue but rather as a cross-cutting theme influencing various aspects of livestock management. This design allowed participants to reflect critically on how gender dynamics shape both individual experiences and community-level outcomes. Joint participation of spouses in the CC sessions created opportunities for men to begin reflecting on restrictive gender norms and cultural perceptions, fostering support for women’s voices and participation. This inclusive approach often led to community-led actions and commitments toward gender-equitable change (Table 4). In Yabello, gender-related discussions were relatively limited due to the short duration of the intervention, which consisted of two rounds of sessions. The dialog primarily emphasized gendered awareness and access to training and information related to animal welfare and antimicrobial use, without delving into deeper issues such as gender roles, decision-making, or underlying normative practices.

**Table 4:** Gender-responsive actions identified by community participants.

Communities	Key actions and commitments
Hawora Arara	Share women’s workload through joint decision-making and mutual support Foster open communication and trust between spouses Promote respect and shared meals as symbols of equality Practice transparency and provide feedback in household roles Challenge and resist social pressures that discourage gender-equitable practices
Ancha Sadicha	Share labor-intensive livestock tasks with women Cooperatively decide and adjust household roles Encourage mindset shifts among both women and men to support male involvement in domestic work Acknowledge women’s knowledge of animal health and commit to including them in livestock decisions
Menz Gera	Reduce women’s workload to enable their participation in meetings and training Provide practical support at home to facilitate women’s access to information Recognize and address barriers that limit women’s involvement in learning opportunities
Menz Mama	Promote gender-equitable attitudes in children by teaching boys domestic skills Support women’s participation in public forums and respect their contribution Share household information and knowledge between spouses Encourage women to join discussions during home visits by extension advisors

Cultural and gender norms appeared to limit women’s representation and participation, particularly during the early stages of the conversations, though this varied across sites. Women in Yabello and Doyogena were more active and empowered during the sessions, whereas in Menz Gera and Menz Mama, restrictive gender norms limited women’s active engagement. Although gender-segregated groups were used in all sites to create safe spaces, age dynamics within women’s groups also shaped participation. Younger women often felt disempowered and hesitant to speak in the presence of older women. To foster inclusive dialog, female local facilitators were engaged in some communities and worked alongside elder women to encourage younger women to actively participate in the discussions.

Participant reflections across sites offered deeper insight into how gender norms and household dynamics constrained women’s access to information and participation in training. A woman participant in Menz Gera shared, ‘When experts make home visits, they speak only with my husband.’ A male participant reinforced this, saying, ‘I usually ask my wife to make coffee during extension visits. I do not invite her to the discussion.’ In Doyogena, women participants identified domestic workload as a major barrier to attending community meetings or training sessions. They also noted that their husbands often refuse to let them participate. One woman explained, ‘My husband attends training, and if I express interest, his refusal keeps me from going.’ A male participant echoed this concern, asking, ‘If women go for training, who will handle the household chores?’ Women also reported that men rarely shared information they received from community meetings or training sessions.

Building on these insights, the conversations created space for participants to share personal experiences, which fostered reflection on cultural constraints and women’s workloads, gradually promoting greater awareness, empathy, and openness to change. Men participants enthusiastically agreed to share domestic work to enable their wives to participate in training and community meetings. A male participant in Menz Mama called on his fellow men to provide women with family support and share domestic activities to free them time to participate in meetings and training sessions. He explained that he participated in hosting guests, stating, ‘When guests come, I help to serve food, and they appreciate that I support my wife.’ He emphasized that he did not feel ashamed of performing domestic tasks, as he was accustomed to them from a young age. He added, ‘Even during my childhood, my stepmother taught me how to do domestic chores. People do not nickname me when they see me engaged in such work.’ Another male participant from Menz Gera described the supportive behavior of his wife following their joint participation in CC sessions. He recounted, ‘She woke up early to prepare breakfast and encouraged me to eat, knowing that I might be away for the entire day.’ Women participants also acknowledged the support they received from men, highlighting that it facilitated their attendance at meetings and training sessions. A woman participant from Menz Gera noted, ‘If I am not at home, my husband can manage the house and the animals. I no longer worry about my household responsibilities when I attend such [meetings and training] events.’ Over time, participants reported increased openness to shared decision-making. During session reflections, about 20% of participants in each community reported making joint decisions with their spouses.

### Multicontextual applications of CCs

In Ethiopia, CCs were used as a participatory method for research, training, planning, and partnership building in livestock management (Table 5).

**Table 5:** Applications of CCs in CRP livestock in Ethiopia.

Application	Description
Participatory research method	Diagnostic research engaged communities in identifying local challenges, and solutions were tested with them to ensure relevance and practical impact.
Participatory training method	Inclusive and experiential learning facilitated collaborative action and problem-solving.
Participatory planning method	An inclusive planning process that centered local voices and fostered ownership
Community-based intervention method	Fostered accountability through joint efforts with service providers and ensured shared monitoring and evaluation
Partnership and engagement method	Community groups were informed and empowered, building demand capacity. Service providers engaged inclusively, strengthening response capacity.

As a participatory research method, the CC approach provided a platform for community members and research partners to analyze challenges and explore context-specific solutions jointly. In Doyogena and Menz, the conversations generated ideas that inspired research partners to collaborate with community groups in testing and developing improved feed management practices. In Doyogena, a socioeconomic researcher reflected on his experience, saying, ‘I previously viewed community members solely as information providers, with minimal feedback or learning for them. Now, I realize this approach [CC] can fulfill both research and learning objectives.’ Similarly, a feed researcher in Menz said, ‘I’ve learned so much from community members about their practices and feed management issues. This [CC] approach helps identify research problems and develop and test technologies with community groups. I now realize how much I’ve missed in making feed research more relevant to communities.’

As a participatory training method, the approach appeared to promote experiential and collaborative learning among communities and local service providers. This participatory dynamic was perceived to make learning more engaging and accessible for both women and men, while potentially enhancing the relevance of the content. Separate gender discussion groups provided safe spaces where women could more comfortably discuss gender norms and practices in livestock management. The process also supported the localization and co-development of learning content, helping to align messages with community perspectives. Moreover, it contributed to strengthening the interaction between community members and service providers. Through this engagement, some local service providers reported gaining new insights into community priorities and improving their communication approaches. A livestock expert in Doyogena reflected, ‘We have only been telling farmers our prescriptive messages, making them only listen to our ideas.’ Similarly, an animal health expert in Menz shared, ‘I usually focused on treatment actions and did not learn from livestock keepers’ perspectives. I now ask them questions to learn about the history of their animals and health management practices.’

In participatory planning and intervention, the CCs fostered joint analysis and intervention planning. The approach provided an effective mechanism for integrating community voices into local planning and intervention processes, ensuring more inclusive and contextually relevant development efforts, while also promoting ownership and inclusiveness. The community actions fostered ongoing engagement, ensuring that service providers continued offering mentoring and problem-solving support, which was essential to sustaining behavior. In Menz Gera, an animal health expert said, ‘it [community dialog] has catalyzed our work’. Similarly, a gender expert remarked, ‘I felt like I was doing my work. Now we [women’s affairs office] have community champions to replicate the experience to other communities.’

As a partnership development tool, the CC approach strengthened engagement and trust-building between communities and service providers, enhancing the ability of community members to seek services while enhancing the capacity of service providers to deliver effective services. Regular sessions created safe spaces for mutual reflection, joint problem-solving, and shared decision-making. In Menz, community groups asked service providers why rabies vaccines were unavailable. The service providers explained that vaccines were available, but users needed to aggregate for accessibility. As a result, communities organized themselves to access vaccination services, bridging the communication gap between them and service providers. This follow-up on community concerns further strengthened credibility and trust.

### CCs as a model for social and behavioral change

Community conversations were used as a structured, participatory model for facilitating social and behavioral change in livestock management. Aligning with behavior change stages, these processes evolved from a confirmatory view of community members to a more critical view of perceptions and practices, to visioning and actions (Table 6).

**Table 6:** Promoting behavior change in livestock management through CCs.

Stages of behavior change	Characteristics of participants	Learning processes
Unawareness	Denial, ignorance of problem	Exploration, analysis, and critical reflection Re-thinking, questioning perceptions and practices
Awareness	Mixed feelings, contradictions, and realization	Identification and analysis of barriers and benefits Introduction of new information and knowledge
Change motivation	Conviction, change desire, and preparation to change	Action messages, information on correct use, knowledge reinforcement, community actions, strategies for sustained engagement
Practicing new behavior	Experimentation with community actions	Action-learning-sharing groups, mentoring, problem-solving support
Behavior maintenance	Influence others and advocate for change through behavior demonstration	Promotion, knowledge sharing, change storytelling, social networks

Initially, participants were generally unaware of the issues in question, adhering to existing norms, structures, and practices. They often described an idealized version of the issues and denied the problems. Many claimed that livestock management and domestic tasks were equally shared between women and men and that both participated equally in household and community decision-making. However, through deep and critical dialogs, facilitated by probing questions and storytelling, participants engaged in self-reflection, re-evaluated their perspectives, and gained new insights into the issues.

Women participants challenged the claim of equal workload distribution by citing their heavy labor burden. This prompted male participants to critically reflect, gradually acknowledging that cultural norms restricted their involvement in traditionally female-designated tasks. For example, in Doyogena, initially, some participants painted an optimistic picture, insisting that men participated in tasks traditionally assigned to women. However, others challenged this view, pointing to persistent cultural barriers to an equitable division of labor. One male participant mentioned taking ‘*Kocho’* (processed food from false bananas) to the market as evidence of his involvement in domestic activities. Kocho was traditionally a woman’s domain, with men discouraged from its processing and marketing. Another male participant countered, saying, ‘While you handle the selling, your wife is primarily responsible for the processing.’ Other participants further questioned the sincerity of his claim, saying, ‘Why do you take the Kocho to Hosanna instead of selling it here in Doyogena? Maybe it’s because you do not want your neighbors to see you doing it.’ Through probing and storytelling, participants became more open and critical of their perspectives. As trust grew, they engaged in open and deeper discussions, questioning and challenging their beliefs and practices.

Awareness and motivation for change were key moments of the CC process when community members recognized the issues and became open to exploring the benefits and challenges of change. As new information challenged existing perspectives and practices, participants often felt conflicted. Building their confidence and readiness for change was crucial, as it involved evaluating the advantages of adopting new behaviors. For example, in Menz Gera, a male participant initially resisted taking on domestic roles, viewing them as culturally reserved for women. However, through critical questioning, he recognized his misconception and expressed a willingness to change. He said, ‘Once, my wife went to visit her relatives and did not return that day as I had expected. I always thought caring for the chickens was her job, so I did not look after them, and that night, predators ate them all. That moment made me realize how my inaction affected the whole family. I now see the importance of sharing domestic responsibilities and have committed to doing my part at home.’ Testimonies from other participants strengthened his confidence and commitment to change. Addressing gaps and doubts, new knowledge was introduced alongside key messages that enhanced understanding and guided the identification of community-driven solutions. Emphasizing the benefits of specific actions further motivated participants to implement these solutions.

Practicing change and behavior maintenance aligned with the action-learning and influencing stage of the CC process. This stage involved community action implementation, problem-solving support by local service providers, and social influence through knowledge sharing and demonstration of new behaviors. To ensure the sustainability of these behaviors, communities of practice were established among local service providers, and capacity-building support was provided to integrate the approach into institutional systems.

### Customization of the CC approach

A dynamic and evolving approach to CCs, shaped by ongoing learning and adaptation during implementation, led to the development of a structured process that facilitated participatory learning and engagement (Table 7). The sessions focused on experiential and collaborative learning between communities and local service providers to explore issues, introduce new knowledge, and co-develop solutions through interactive dialogs. By embedding reflection, action planning, and follow-up strategies, the process enhanced the sustainability of learning and practice change. Process documentation captured behavioral shifts, reinforcing the importance of continuous engagement, institutional support, and community ownership in driving long-term social and behavioral transformation.

**Table 7:** Adapted CC process as implemented in CRP livestock in Ethiopia.

Phase	Learning activities	Objectives	Methods	Outcomes
Design phase	Assessment and content development	Analyze key community challenges and knowledge gaps Develop dialog content	Focus group discussions Key informant interviews	Problem situation analyzed, discussion content co-developed
	Facilitation team preparation	Select and train local facilitation team	Hands-on facilitation and documentation training	Clear understanding of process and facilitation roles among the facilitation team
	Community entry and mobilization	Secure support from community leaders and local service providers Ensure diverse participation	Representation criteria Participation list	Representation and participation of women, men, and local service providers ensured
	Baseline knowledge, attitudes, and practices (KAP) survey	Establish pre-intervention KAP baseline	Interviews using semi-structured questionnaires	Baseline KAP data collected
Delivery phase	Session opening and context setting	Build rapport and create open discussion environment	Elders’ blessings Scenarios and narrative stories	Safe, engaging space for discussion established
	Exploration and analysis	Explore diverse perspectives, identify knowledge gaps, and stimulate experiential learning	Thought-provoking questions Role-playing Interactive dialog	KAP gaps identified, motivation for learning and change fostered
	Knowledge introduction and reinforcement	Address knowledge gaps, enhance community understanding	Pictorial presentations Interactive discussions Demonstrations	Improved knowledge, new perspectives introduced
	Identification of community actions	Translate learning into action, establish change indicators	Problem-solution tree, action plan matrix	Community actions developed, commitment to implementation fostered
	Reflections, follow-up strategies, and closing	Review learning, assess experiences, and establish monitoring mechanisms	Reflection questions Local partner commitment Follow-up strategies	Follow-up strategies outlined and institutional ownership ensured
Experimentation phase	Community action implementation and influencing through behavior demonstration and informal networks	Peer learning circles Support knowledge application and broader community influence	Problem-solving support Peer learning and information sharing	Practice change facilitated, community engagement sustained
Scaling phase	Outcome documentation Cross-community exchanges	Measure changes in KAP Celebrate success Establish communities of practice	Post-intervention KAP survey, outcome documentation	KAP changes assessed, best practices documented for scaling

The implementation of the CC intervention was marked by deliberate customization to fit the diverse local contexts. Key adaptations included tailoring session schedules to align with community routines, employing local translators to bridge language barriers, and adjusting facilitation methods to accommodate varying literacy levels and cultural norms. Thematic focus areas were adapted to reflect livelihood differences, such as pastoralism versus crop-livestock farming. Crucially, integration with existing local structures appeared to enhance relevance and sustainability. These adaptations highlighted the importance of flexibility and responsiveness, with local facilitators playing a central role in shaping the approach. Continuous feedback and reflection allowed iterative refinement, suggesting that balancing core CC principles with contextual adaptation could enhance engagement and community ownership.

## Discussion

Conventional approaches mainly focus on information dissemination, often leaving little room for joint analysis, collaborative learning, and action (4). In contrast, participatory approaches enable practitioners to engage directly with communities, understand their perspectives, and co-develop solutions (35). These approaches have gained increasing attention for their ability to ground interventions in local realities, fostering community-driven solutions (10, 36).

This study demonstrates the versatility of the CC approach in facilitating participatory engagement in livestock management. The approach provides an inclusive platform for communities to engage in participatory discussions, share knowledge, and co-create solutions. Similarly, in forest management, the approach has been shown to support locally appropriate decision-making, participatory democracy, and sustainable solutions (37). Unlike conventional meetings and training sessions, which often exclude women (38), the CC approach takes place in convenient settings, making it more accessible to diverse community participants. The approach recognizes that adults learn best in environments that are socially and culturally meaningful to them (39), ensuring that both women and men can actively engage in discussions on pertinent community issues.

The CC intervention, conducted between 2018 and 2019, differed across communities in both duration and thematic scope, which shaped the depth and outcomes of the dialog processes. In Doyogena and Menz, the intervention took place in 2018 and comprised four rounds of discussions over approximately 6 months. Held initially at monthly intervals and spaced out later, these sessions addressed topics including gender roles, division of labor, zoonotic diseases, ownership, mobility, and decision-making in livestock systems. This extended and repeated engagement created space for both women and men participants to build trust with facilitators and each other, enhancing their confidence, openness, and social cohesion. The structured design of the four-round CC process in Doyogena and Menz supported progressive learning and deeper engagement. The first round typically introduced key issues and promoted awareness-raising and problem identification. In the second round, discussions moved toward examining underlying social norms, constraints, and enablers. By the third round, increased participant familiarity with the process allowed for co-identification of practical, context-specific actions and gender-responsive solutions. The final round consolidated learning, captured participant stories and reflections, and gathered feedback on how new knowledge was integrated into daily life. This phase also provided insights into perceived changes in attitudes or practices and reinforced a commitment to sustained action. The monthly spacing between sessions was critical in enabling participants to reflect, experiment, and return with experiences that enriched subsequent conversations.

In contrast, the CC intervention in Yabello consisted of two rounds of discussions, focused on animal welfare, antimicrobial use, and antimicrobial resistance. Due to its short duration, there was limited opportunity to observe evolving patterns of interaction over time. Gender-related discussions in Yabello were comparatively narrower, emphasizing gendered awareness and access to training and information related to animal welfare, antimicrobial use, and antimicrobial resistance, rather than exploring roles, decision-making, or normative practices. This contrast highlights the importance of intervention design, particularly duration, frequency, and thematic breadth, in influencing the depth and effectiveness of gender-responsive dialogs. The sustained engagement and thematic layering in Doyogena and Menz enabled a more transformative learning process and the emergence of community-defined gender actions, which were less feasible in the shorter engagement in Yabello.

Alongside promoting dialog and knowledge sharing, the CC process appeared to support gradual shifts in social learning and behavior. The approach creates safe spaces for critical dialogs and allows community participants to examine restrictive gender norms, attitudes, and practices. Studies have shown that participatory learning processes help communities critically reflect on gender roles and household decision-making dynamics (31, 37). This study aligns with those findings, showing that CCs contribute to shifts in gender relations by encouraging households to discuss and negotiate decisions together. Empirical evidence suggests that couple discussion groups addressing economic issues and gender norms significantly enhance cooperation, decision-making, and equity at both household and community levels (40). Behavioral reinforcement theories further support this, indicating that behaviors reinforced through positive experiences are more likely to be sustained (41). Households became arenas of cooperation (42), where both spouses benefited from the conversation outcomes. As a result, men in the communities became more willing to collaborate with their spouses, as household cooperation positively reinforced their behavior.

The CCs also foster relationship-building between community members and local service providers, enhancing communication and co-generation of solutions. When community members and animal health professionals engage in mutual learning, they develop a deeper understanding of each other’s perspectives, leading to more responsive and effective services (43). Studies suggest that participatory approaches strengthen trust, collaboration, and long-term community ownership (28). Informal conversational networks formed through CCs serve as vehicles for social transformation, allowing information and ideas to spread organically across communities.

The value of the CC approach depends on the quality of its design, facilitation, and documentation process. Effective CCs require skilled facilitation, reflective writing, and thorough process documentation. Rich documentation and high-quality facilitation are essential for successful implementation. Immediate reflection, review, and summarization after each session are critical to capturing learning and outcomes (28). Documentation should take the form of a ‘thick description’, making it an integral part of the CC process.

The inclusion of a cross-section of community members contributed to the richness and diversity of perspectives in the CC sessions. This heterogeneity enhanced the relevance and contextual grounding of the discussions, particularly around socially embedded issues, such as gender norms and decision-making in livestock management. While the study did not aim to quantitatively measure the differential effects of participant categories, observational and qualitative data suggest that the diverse composition of participants facilitated mutual learning and more inclusive dialog. For example, the presence of local leaders and service providers helped link community discussions with existing institutional structures, while the inclusion of youth and women, particularly those from male-headed households, helped surface perspectives and needs that are often underrepresented in public forums. This diversity contributed to the identification of context-specific gender-related actions.

Initially, participants anticipated that facilitators would ‘teach’ them in a conventional, top-down manner, reflecting their prior experiences with local service providers who typically delivered information through directive instruction. This made open discussion and collaborative learning challenging at first. It was therefore essential to clarify from the start that the sessions were not traditional training sessions, but spaces for dialog and shared learning. Active methods, such as using pictures to spark conversation, helped set the tone and encouraged participants to engage and share more actively. Over time, participants gradually embraced this approach, developing the attitudes, skills, and confidence needed to collaborate effectively among themselves and with local service providers to address their concerns (28). Although initially challenging, the CC method fosters sustained community empowerment and engagement.

Sustaining CC outcomes requires integration with local service structures. This was facilitated through the deliberate alignment of CCs with existing local planning and intervention frameworks. From the outset, local service providers were trained as facilitators and actively involved in co-developing conversation materials, helping to embed the approach within routine service delivery mechanisms. Community ownership was fostered through the active involvement of local leaders, elders, and cooperative members, while informal follow-up groups emerged in some areas to monitor progress on agreed actions. This study highlights the importance of developing the engagement capacity of both community members and local service providers to support long-term improvements. Embedding community voices into local planning ensures that interventions remain community-driven and responsive to evolving needs. According to self-determination theory, behavioral change is more likely to be sustained when community members have a sense of autonomy and a clear understanding of the relevance of new behaviors (44). Aligning community-led actions with local service support structures can enhance the long-term impact of CCs as a model for social and behavioral change.

## Limitations of the study

The study underscores the potential of CCs as a flexible and context-sensitive approach for fostering inclusive dialog, community learning, and gender-responsive change in livestock systems. However, it is not without limitations. The findings are based primarily on qualitative reports, anecdotal accounts, and participant testimonies, which may be subject to social desirability bias. Reported behavioral changes were not independently verified through longitudinal tracking. A more rigorous quantitative assessment would provide stronger empirical evidence and allow for comparative analysis across sites. Future applications of the CC approach would benefit from incorporating pre-and post-evaluations to assess impact over time, along with the use of qualitative analysis tools such as NVivo to enhance the depth and rigor of data analysis. Further research is also needed to explore how differences in participant composition influence outcomes across contexts. Despite these limitations, the study contributes to the growing body of evidence on participatory learning and action methods. The findings support existing literature on the role of CCs in fostering community engagement, shifting social norms, and co-developing locally relevant solutions. Mixed-method research designs would strengthen future evaluations and provide a more comprehensive understanding of long-term outcomes.

## Conclusion

Community conversations not only help identify and analyze pertinent community issues but also foster community-led actions in collaboration with service providers. Through joint analysis, mutual learning, and collective decision-making, the approach has shown potential to strengthen communication and enhance understanding between community members and service providers. It provides an inclusive platform where diverse perspectives, particularly those of women and men community members, can be expressed, fostering a sense of ownership and accountability over community actions. While initially challenging, trust-building and sustained participation in CCs can help strengthen the confidence, skills, and attitudes that support collaboration and problem-solving between communities and service providers. Over time, incremental self-reflection and awareness contribute to shifts in knowledge, attitudes, and practices within communities. Through this process, community members develop greater self-awareness and empowerment, enabling them to take informed action at both the household and community levels.

## Data Availability

The datasets analyzed in this study are published as reports in institutional repositories and are cited in the reference list.
